# Supplementary material for: DeepDive: estimating global biodiversity patterns through time using deep learning
Source: Nat Commun. 2024 May 17;15:4199. doi: 10.1038/s41467-024-48434-7 (PMC11101433; doi:10.1038/s41467-024-48434-7)
Supplement: Supplementary file 1 — Supplementary Information [file 41467_2024_48434_MOESM1_ESM.pdf]

Supplementary Materials for

DeepDive: Estimating global biodiversity patterns through time using deep learning

Rebecca B. Cooper[1,2], Joseph T. Flannery-Sutherland[3], Daniele Silvestro[1,2,4]

1. Department of Biology, University of Fribourg, 1700 Fribourg, Switzerland,

2. Swiss Institute of Bioinformatics, 1700 Fribourg, Switzerland,

3. School of Geography, Earth and Environmental Science, University of Birmingham, Birmingham, UK,

4. Department of Biological and Environmental Sciences, Global Gothenburg Biodiversity Centre, University of Gothenburg, Gothenburg 413 19, Sweden

Correspondence: rebecca.cooper@unifr.ch, daniele.silvestro@unifr.ch

Supplementary Figures

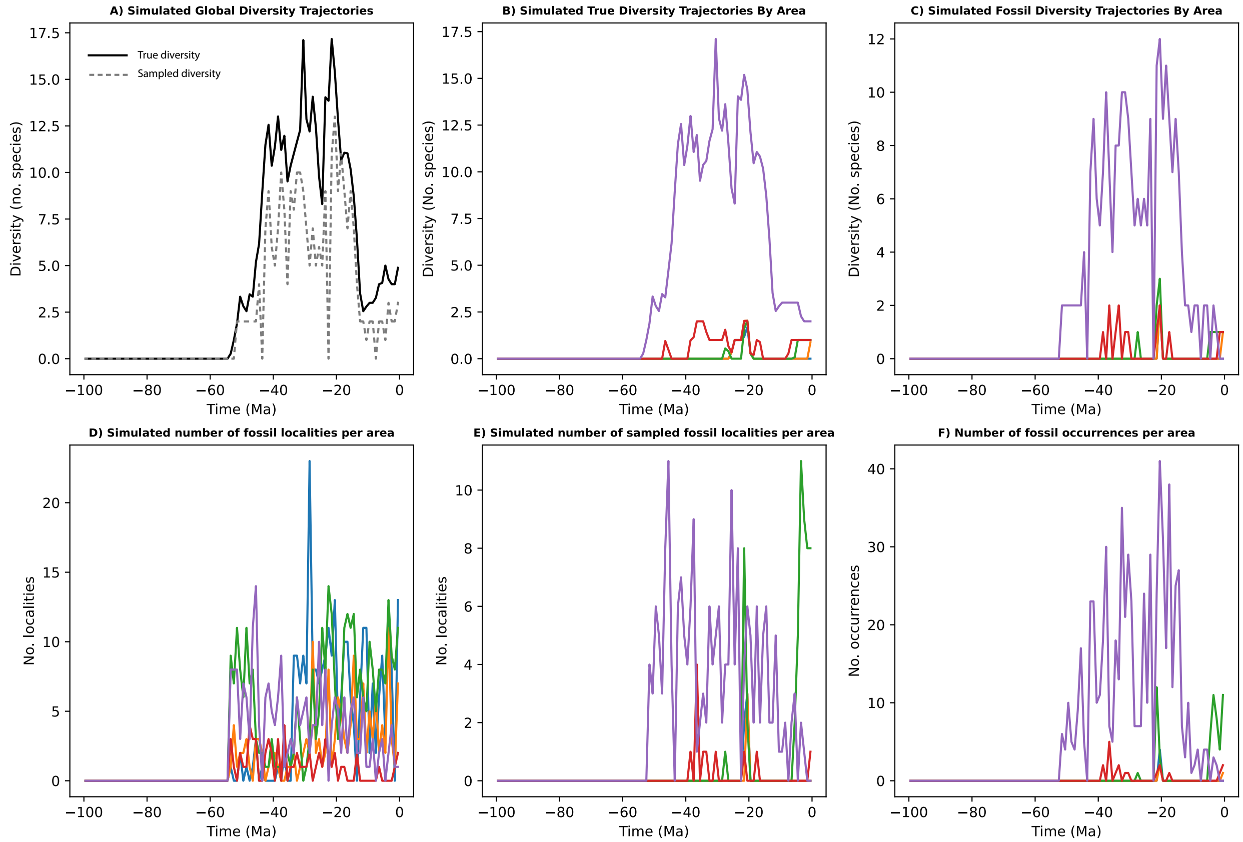

Supplementary Figure 1. Example of a DeepDive simulation. Where A) shows a simulated diversity trajectory and sampled trajectory, B) a regional breakdown of simulated diversity curves, C) a regional breakdown of sampled diversity curves, D) the number of localities per region, E) the number of localities per region which are sampled and F) the number of fossil occurrences per region. In all cases except A) different colours indicate different regions.

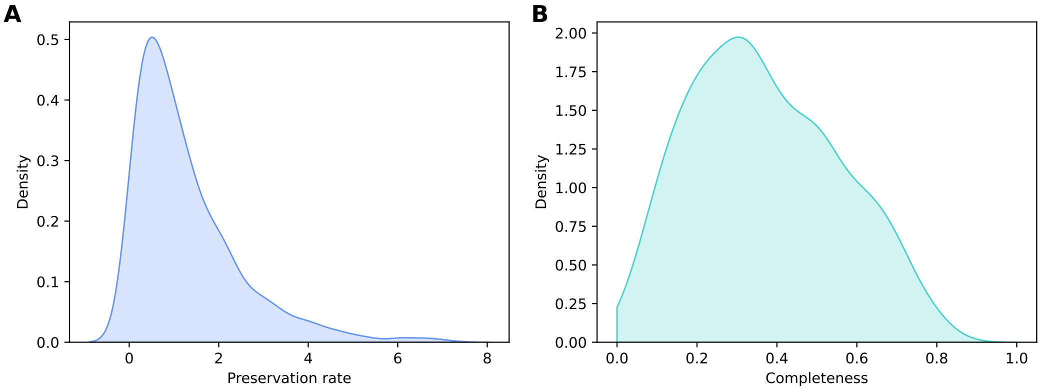

Supplementary Figure 2. Distribution of A) preservation rate (expected number of fossil occurrences per lineage/time unit) and B) completeness (fraction of sampled lineages in the fossil record) for 1,000 simulated test datasets.

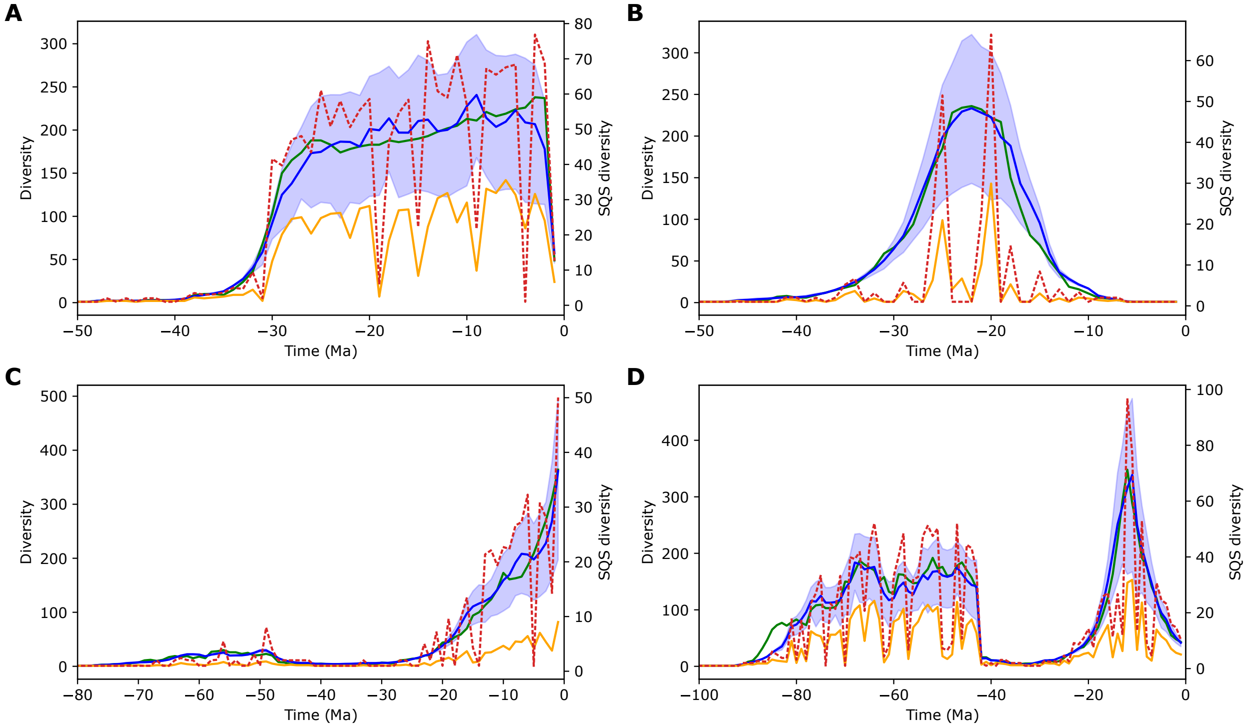

Supplementary Figure 3. Examples of various potential biodiversity trajectories through time (Ma), where the simulated diversity (green) is plotted relative to the DeepDive estimation with confidence intervals (blue), face-value fossil counts (orange) and SQS at quorum = 0.6 estimated diversity (dashed red). Trajectories include examples of A) times of stability and mass extinctions, B) the rise and fall of clades, C) low, stable diversity and radiation, D) episodes of mass extinction and recovery.

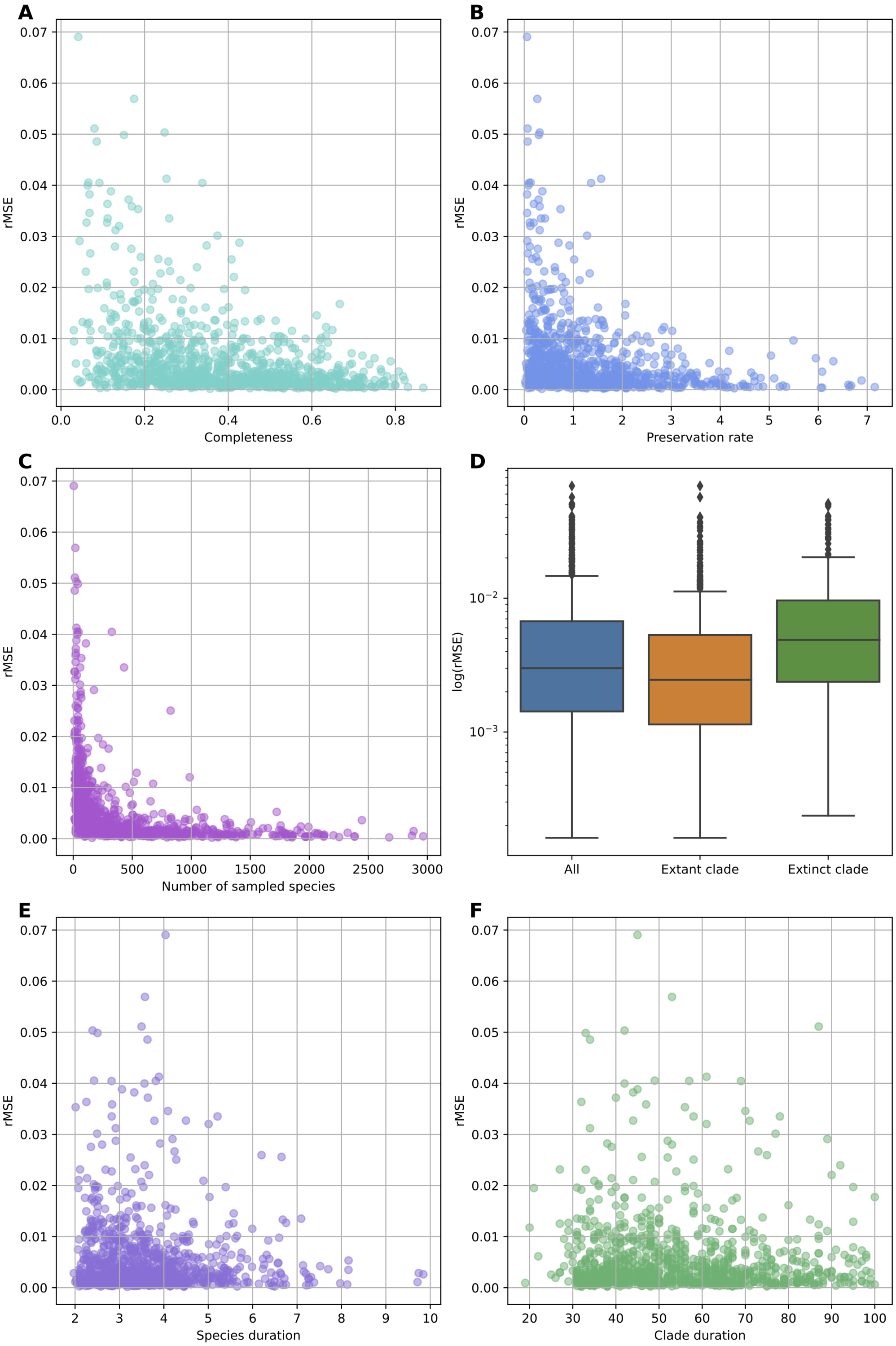

Supplementary Figure 4. Variation in relative MSE for the fit of DeepDive diversity estimates from simulations to actual global diversity within those simulations with A) completeness (proportion of simulated species sampled as "fossils"), B) preservation rate, C) number of sampled species, D) extinct or extant state of clade, E) species duration and F) clade duration for 1,000 simulated test datasets.

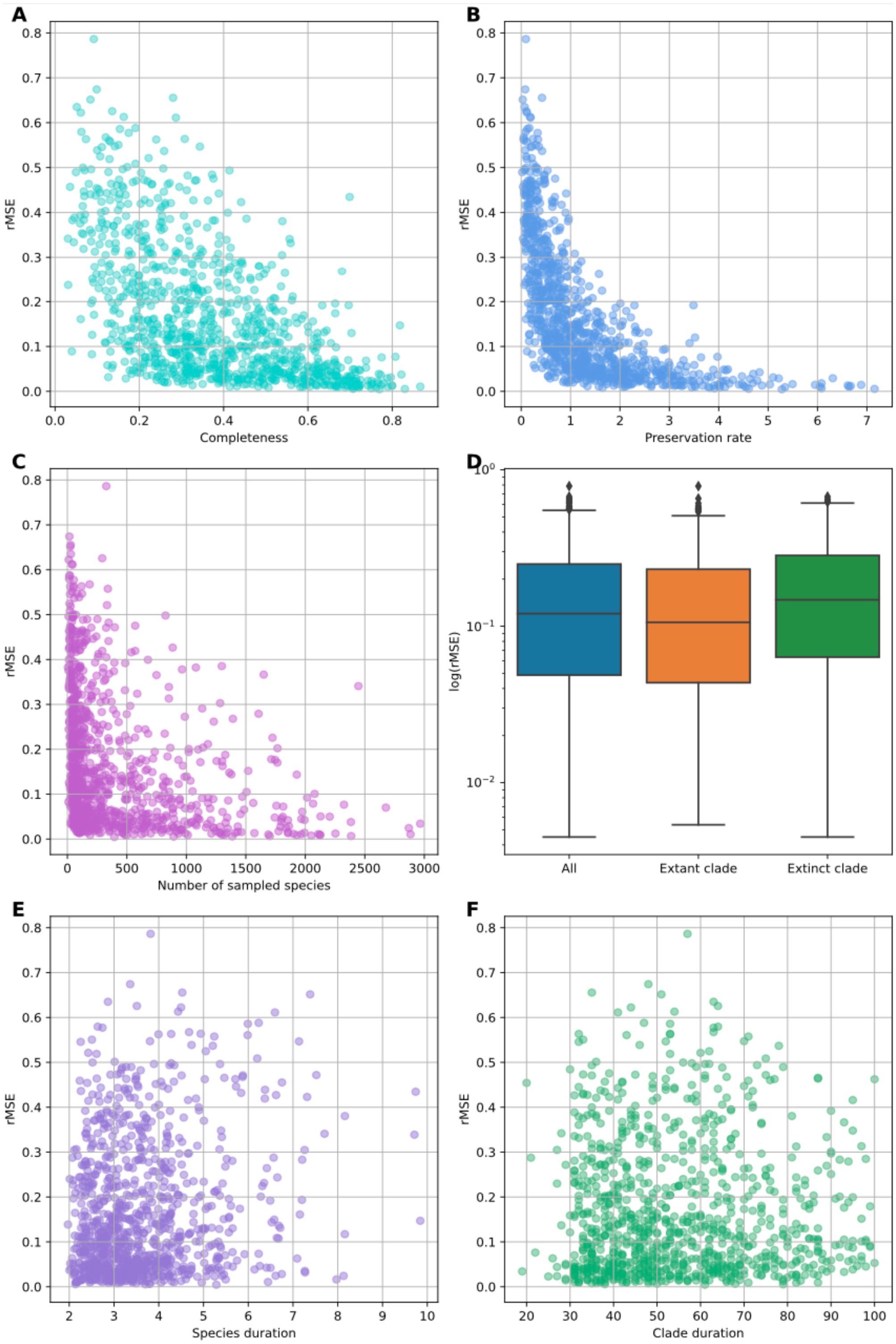

Supplementary Figure 5. Variation in relative MSE in SQS estimates with A) completeness, B) preservation rate, C) number of sampled species, D) extinct or extant state of clade, E) species duration and F) clade duration for 1,000 simulated test datasets.

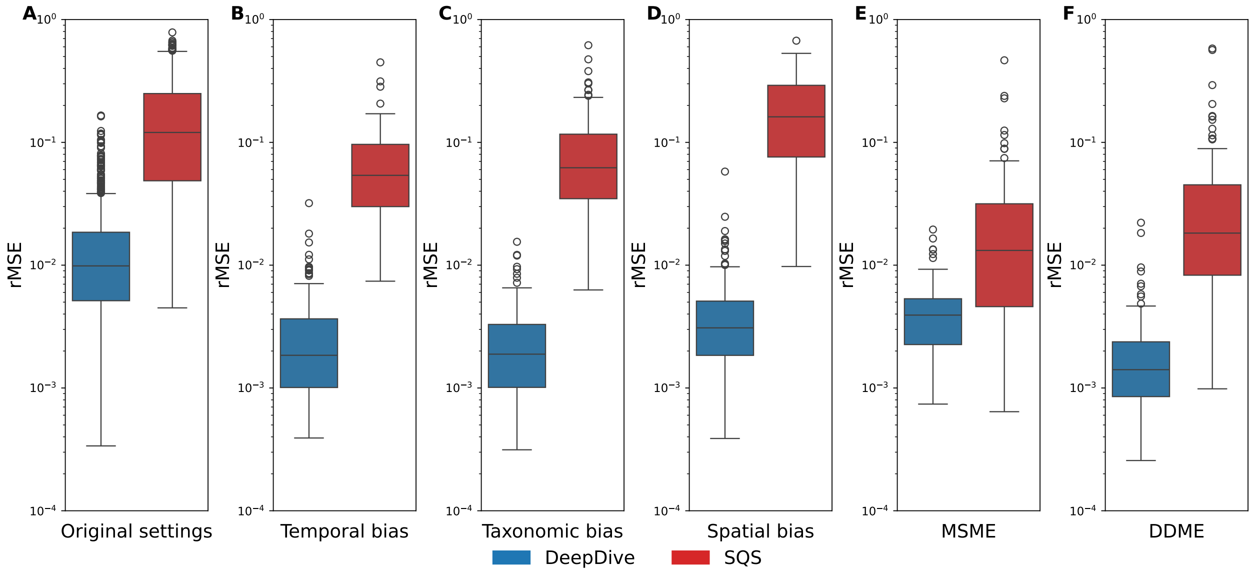

Supplementary Figure 6. Relative MSE of biodiversity estimates using a DeepDive trained model that contains additional patterns (mass speciations and mass extinctions, and diversity dependence followed by mass extinction) that were rare in the training set of the original model for A) a test data set generated under the same parameterisation as the original DeepDive trained model, and for test sets generated under different parameterisations to represent conditions of strong B) temporal, C) taxonomic and D) spatial biases along with patterns that were rare in the training set of the original model E) mass speciations and mass extinctions, and F) diversity dependence followed by mass extinction for DeepDive and SQS. Data are presented as median values +/- the interquartile range, whiskers at 1.5 IQR. n = 100,000 (100 time bins x 1,000 simulations).

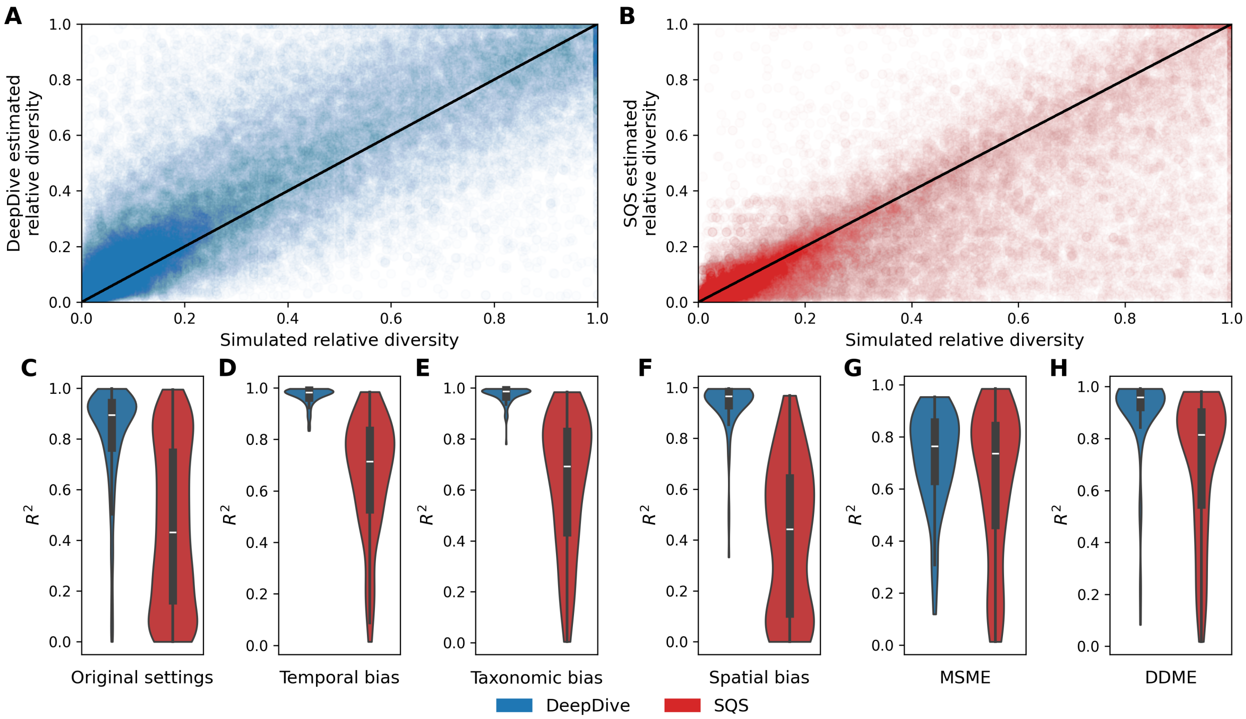

Supplementary Figure 7. Accuracy of biodiversity estimations relative to simulated diversity for test datasets where A) shows estimates made using a re-trained model in the DeepDive approach including patterns that had previously been rare in the training set (mass speciations and extinctions, diversity-dependence followed by mass extinction) and B) SQS at quorum level 0.6, in both cases the black line of slope 1 indicates the goal of these methods to make as close to a 1:1 estimate as possible. The variation in R^2 C) for test datasets and variation in R^2 for test sets generated to represent conditions of strong D) temporal, E) taxonomic and F) spatial bias and for patterns that were previously rare in training simulations G) mass speciation and mass extinctions, H) diversity dependence followed by mass extinction (see Methods for more details) for DeepDive and SQS. Data are presented as median values +/- the interquartile range, whiskers at 1.5 IQR. n = 100,000 (100 time bins x 1,000 simulations).

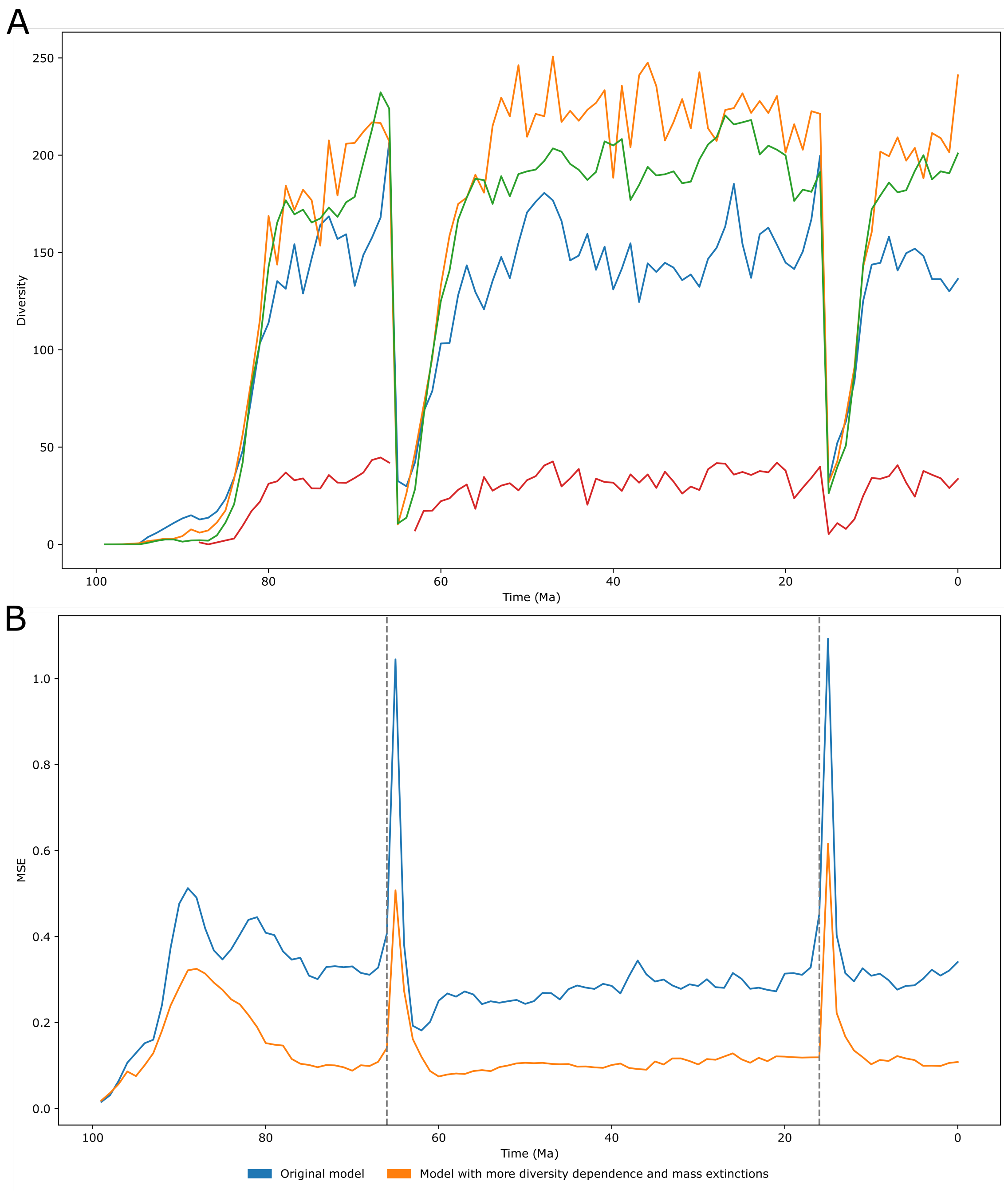

Supplementary Figure 8. A) Simulated diversity (green) and DeepDive estimates using the original model (blue) and a model trained with inclusion of diversity dependence followed by mass extinction patterns in the training set (orange) with SQS estimates (quorum 0.6, red). B) The error (MSE) associated with estimates using the DeepDive trained model spikes in the time bin following mass extinction events (dashed lines) before recovering quickly. A model including DDME patterns in the training set demonstrates lower spikes in error at these events.

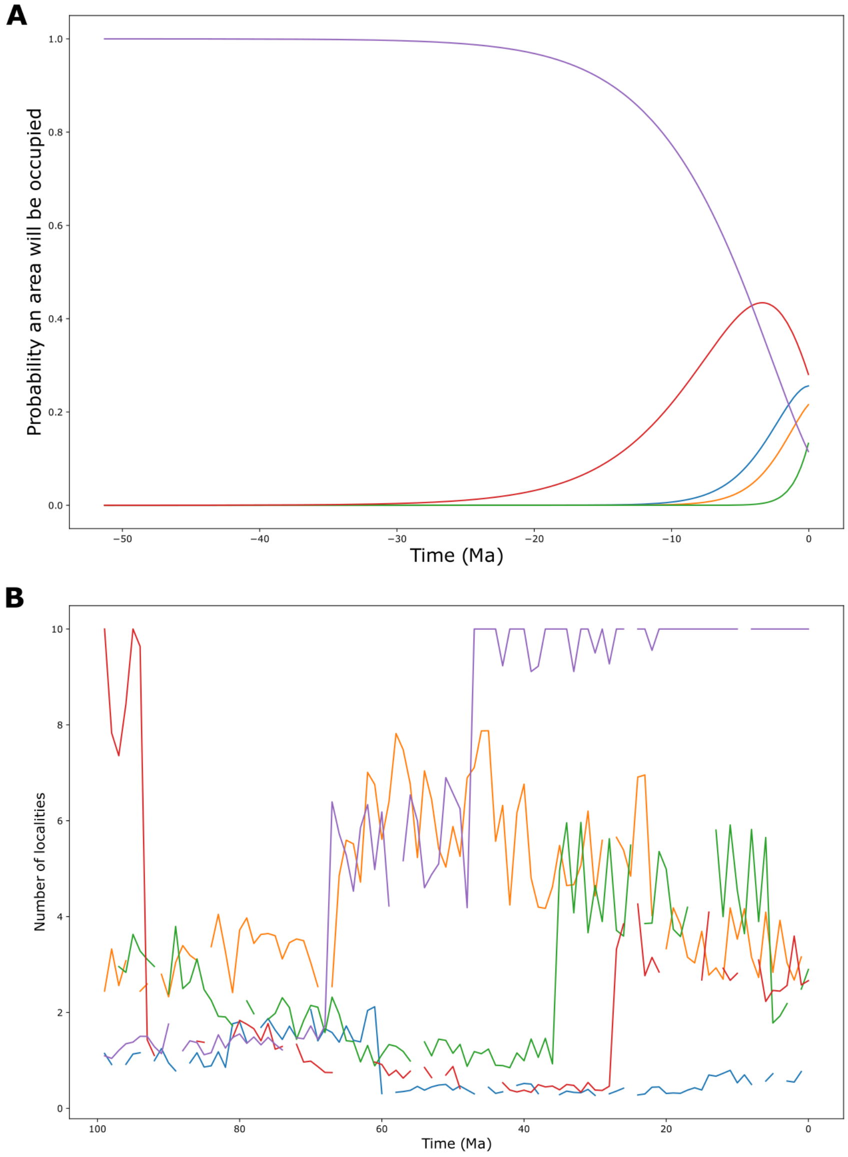

Supplementary Figure 9. A) Example probability that a species initial range will originate in a region (blue, green, orange, purple or red) through time (Ma). This in our notation is indicated as $\kappa_1(t), ..., \kappa_A(t)$ [Eqn. 3] where κ is the relative carrying capacity. B) An example of the number of localities drawn per region through time where each line represents a different region for one simulation.

Supplementary Tables

Supplementary Table 1. Variables used within the DeepDive simulator and their notation.

Notation	Explanation
p_{ME}	Probability a mass extinction will occur per ma
μ_{ME}	Extinction probability for a given time bin
A	Number of discrete geographic regions
α	Concentration parameter that describes how similar regions are in size
R_s	Initial regions a species s occurs in
κ	Ability of a region to host a species
k	Constant linking region sizes to their probability to host a species
s_i	Speciation time of a given species
c_a	Slope describing how carrying capacity changes through time in a region a
w_s	Species-specific dispersal rate
δ_{ij}	Vector of relative distances between regions
d_ij	Distance matrix
$P(j\|i)$	Probability a species will occur in other regions
r_a	Region-specific sampling rate of fossiliferous localities for a region a
d_a	Relative size of a region a
α_r	Shape parameter of a gamma distribution which determines r_a
β_r	Rate parameter of a gamma distribution which determines r_a
ζ	Slope of the mean sampling rate through time
q_t	Sampling rate of fossiliferous localities at a time t
p_{gap}	Probability of a gap in the fossil record for a given time bin
z_{at}	Occurrence of sampling gap in region a and at time t
η	Median from which region and time specific random effects are drawn
ε_{at}	Time and region specific random effects
m	Multiplier which introduces stochasticity drawn from a uniform distribution
b	Median of the distribution for multiplier m
Δ_t	Time bin duration
λ_{at}	Expected number of localities for each region and time bin
l_{at}	Number of fossil localities in a region in a time bin
ψ_s	Species specific preservation rate - expected number of fossils of a given species s per locality
p_s	Probability a species is recorded in a locality
S_{at}	Number of species living in region a at time t
f_{at}	Number of sampled occurrences representing the fossil record in a region a at time t across a set of species S_{at}

Supplementary Table 2. Model testing. Variation in the validation MSE scores for DeepDive trained models across varying architectures. Long short term memory units (LSTM) of 1 layer (32 nodes) or 3 layers (128, 64, 32 nodes). Dense node layers not fully connected or fully connected with 2 layers (64, 32 nodes). Drop out fractions of 0.05 or 0.1.

LSTM layers	Fully connected	Dropout	Validation MSE	Training loss	Number of training epochs	Test MSE
1	N/A	0.05	0.132	0.125	229	0.229
1	N/A	0.10	0.132	0.130	141	0.223
1	2 layers	0.05	0.119	0.110	134	0.207
1	2 layers	0.10	0.117	0.114	127	0.206
3	N/A	0.05	0.114	0.106	18	0.199
3	N/A	0.10	0.115	0.105	19	0.203
3	2 layers	0.05	0.115	0.106	19	0.200
**3**	**2 layers**	**0.10**	**0.114**	**0.107**	**18**	**0.197**

Supplementary Table 3. Permo-Triassic marine-like model validation. Variation in the validation loss, training loss and number of training epochs used for Permo-Triassic marine-like models across varying architectures and test accuracy. Long short term memory units (LSTM) of 1 layer (32 nodes), 2 layers (64, 32 nodes), 3 layers (128, 64, 32 nodes) or 4 layers (256, 128, 64, 32 nodes). Dense node layers not fully connected or fully connected with 1 layer (32 nodes). Drop out fraction of 0.05.

LSTM layers	Fully connected	Dropout	Validation loss	Training loss	Number of training epochs	Test MSE
1	N/A	0.05	0.186	0.184	52	0.184
1	1 layer	0.05	0.186	0.187	28	0.181
2	N/A	0.05	0.178	0.183	25	0.184
2	1 layer	0.05	0.178	0.178	22	0.179
3	N/A	0.05	0.174	0.173	18	0.172
3	1 layer	0.05	0.178	0.187	13	0.177
4	N/A	0.05	0.175	0.195	11	0.181
4	1 layer	0.05	0.175	0.169	14	0.184

Supplementary Table 4. Proboscidea-like models validation. Variation in the validation loss, training loss and number of training epochs used in training models across varying architectures and test accuracy. Long short term memory units (LSTM) of 1 layer (32 nodes), 2 layers (64, 32 nodes), 3 layers (128, 64, 32 nodes) or 4 layers (256, 128, 64, 32 nodes). Dense node layers not fully connected or fully connected with 1 layer (32 nodes). Drop out fraction of 0.05.

LSTM layers	Fully connected	Dropout	Validation loss	Training loss	Number of training epochs	Test MSE
1	N/A	0.05	0.151	0.154	24	0.147
1	1 layer	0.05	0.151	0.186	21	0.147
2	N/A	0.05	0.141	0.135	31	0.139
2	1 layer	0.05	0.141	0.152	21	0.137
3	N/A	0.05	0.139	0.140	12	0.137
3	1 layer	0.05	0.141	0.146	15	0.138
4	N/A	0.05	0.140	0.139	12	0.137
4	1 layer	0.05	0.140	0.147	13	0.137
